# Do socio-cultural practices by elderly women influence obstetric complications? A study in Limpopo province

**DOI:** 10.4102/curationis.v47i2.2636

**Published:** 2024-12-20

**Authors:** Langanani C. Makhado, Ndidzulafhi S. Raliphaswa, Mary Maluleke, Mutshinyalo L. Netshikweta

**Affiliations:** 1 Department of Advanced Nursing Science, Faculty of Health Sciences, University of Venda, Thohoyandou, South Africa

**Keywords:** complications, elderly women, obstetric practices, pregnant women, socio-cultural

## Abstract

**Background:**

Worldwide, women continue to die from obstetric-related complications, despite the global progress made to reduce maternal mortality. Elderly women play a key role in using their own socio-cultural practices during pregnancy and childbirth.

**Objectives:**

The study aimed to explore the practices based on the beliefs of elderly women in Limpopo province.

**Method:**

A qualitative approach using descriptive, explorative and contextual design was employed. Audio recording of unstructured focus group discussions was conducted of the elderly women who were purposively sampled. Data were analysed and coded using thematic analysis approach following Tesch’s method and co-coded to ensure trustworthiness. Ethical considerations were also adhered to.

**Results:**

The findings discuss practices based on beliefs of elderly women and the need for them to be trained to participate in maternal health practices.

**Conclusion:**

The study concluded that practices based on beliefs of elderly women lead to delays in seeking medical intervention. Therefore, elderly women need to be trained to reduce maternal mortality.

**Contribution:**

This study adds knowledge to the need for training and the importance of elderly women to be trained in order to reduce maternal mortality.

## Introduction

Although the survival of both the mother and baby during pregnancy and childbirth is universally important, the celebration of the baby at birth can evoke feelings of excitement and joy as well as ambiguity and scepticism compared to the survival of the mother. In agreement with Wojtkowiak (2020), the arrival of a new member of the community has traditionally been marked by traditions, as a result, birth is regarded as a person’s first significant life event. Some of the most amazing ceremonies in the world are still associated with childbirth; however, this does not guarantee that all communities across the globe celebrate each and every new birth (Rana & Pandya 2022). The death of the mother during pregnancy and child birth is a serious public health concern; therefore, well-known causes of maternal mortality have been categorised broadly as medical and socio-cultural (Yarney 2019). From a social and cultural point of view, elderly women are stakeholders and caregivers with the potential to influence pregnant woman (PW) from the period of conception until term, and again at postnatal period to the breastfeeding mothers (BFMs) at home. Therefore, they play a key role during pregnancy and childbirth at the community level and have influence in decision making regarding the care of pregnant women using their own indigenous knowledge. Indigenous knowledge is the knowledge that one acquires from their own experience or through apprentice of elders (Dlamini 2024). In support of this, El Hajj and Lone Holst (2020) alluded that the major reason for using herbal medicines among PW was the belief that it was safe to do so, and it was influenced by elderly women. In South Africa, 50% of women reported that they still consult traditional birth attendants as their first option for prenatal care, labour and delivery care and postnatal care, despite the availability and accessibility of mother and child health facilities (Seopa 2021).

In agreement, Awolayo (2019) states that cultural practices have an impact on women’s lifestyle, maternal health decisions and the use of healthcare services; however, all these factors are influenced by cultural practices of elderly women as caregivers of pregnant women at home.

The main causes of death accounting for 75% of maternal deaths are preventable diseases which include: haemorrhage, hypertensive disorders, infections, obstructed labour and unsafe abortion (eds. Macdonald & Magill-Cuerden 2017). However, socio-cultural factors including low socio-economic status of women, dietary practices, taboos surrounding pregnancy and the use of untrained traditional health practitioners (THPs) may also contribute to maternal and child morbidity and mortality (Nisha et al. 2021). Maternal mortality remains a global challenge to the health care system till date. An estimation of about 303 000 women died following pregnancy and childbirth especially in low-income resource countries and most of these deaths were probably preventable (Okonofua 2021). The death of a pregnant women is reported nearly every 8 min globally, either from complications during pregnancy, labour or postnatal period (Owen, Cassidy & Weeks 2021). Sub-Saharan Africa accounts for the majority of maternal deaths worldwide, most of which are caused by a lack of access to prenatal care and skilled birth attendant during delivery (Ahinkorah et al. 2021). In South Africa approximately 20% of maternal deaths occurs outside healthcare facilities and the cause of death is almost the same as cause of maternal death in healthcare facilities (Saving Mothers 2017). Even though there was a decrease in maternal mortality rates from 150 per 100 000 live births to 113 per 100 000 live births in 2019, there was an increase between 2020 and 2021 because of the lack of human and material resources (Mahada, Tshitangano & Mudau 2023).

Out of the nine provinces in South Africa, Limpopo, Free State, Eastern Cape and KwaZulu Natal are said to have the highest rates of maternal fatalities. Limpopo province was classified under the province with highest number of maternal deaths between 2020 and 2021 counting at 165/1000 live births (Massyn et al. 2018).

Cultural practices of elderly women affect PW on when to initiate antenatal care (ANC) and seek medical assistance during labour and postnatal care (Chimatiro et al. 2018; Nyondo-Mipando et al. 2023). According to Warri and George (2020) and WHO (2016), women should start to book for ANC as early as less than 12 weeks of gestation, deliver at the healthcare facility where there are skilled birth attendance and attend postnatal visit at the healthcare facility in day three post-delivery and again at 6 weeks’ time to identify health risk and provide proper management as measure of improving maternal health. In addition, Tesfaye et al. (2020) and WHO (2016) reveal that there are a number of factors that prevent women from seeking health care services during pregnancy, labour and postpartum which include poverty, distance travelled, the lack of information, inadequate services and cultural practices. This means that a solution for these barriers has to be investigated in order to improve maternal health and good perinatal outcome.

Health programmes and strategies developed for the reduction of maternal mortality focussed mainly on biomedical cause whereas non-biomedical causes such as socio-cultural factors and traditional beliefs are not attended to even though they are considered as the root cause of most of maternal mortality (Awolayo 2019). Most of the studies highlight the issue of providing health education to pregnant women, training of health care provider, involvement of men for support and developing bundles to guide care (Lunda, Minnie & Lubbe 2024; Nyfløt & Sitras 2018; Ragolane 2017).

The South African government has continually done much so far in putting programmes to facilitate quality care improvement of pregnant women and newborn babies which includes the introduction of free services to reproductive health including maternity services.

Despite all of the efforts by the Department of Health, it is still obvious that there are gaps and more work needs to be done as the mortality rate is still very high. In support of this, Buser et al. (2020) and Dehury and Dehury (2018) revealed that there are no government guidelines that address the issue of maternal health and child care specifically that concern the traditional beliefs and cultural practices. Elderly women as caregivers play a significant role in the life of pregnant women and newborn babies at home. Because they do not undergo any formal training hence, they use indigenous knowledge acquired from their own experience or from elders for maternal and child health (Adongo et al. 2024; WHO 2016). Community relies on elderly women to render maternal and childcare especially in most rural villages. From an African point of view, to disregard elders’ opinions will cause a curse over your life. In addition, some studies show that elderly women can act as birth attendants using their own experiences even if they did not undergo formal training (Shimpuku et al. 2021; Siruma, Hornby & Srinivas 2014).

Regardless of the important roles played by these elderly women as viewed by community members, little is known about how they contribute to maternal and child health. To the best knowledge of the researcher, no studies have been conducted in Limpopo to address the roles of elderly women in participation in maternal health practices nor any training offered to them regarding maternal and child health. Therefore, this study sought to understand and evaluate if socio-cultural practices by elderly women influence obstetric complications among pregnant women in Limpopo province and develop training programme for elderly women in participation to maternal and child health that might assist in the reduction of maternal mortality.

### Aim

The purpose of this study was to understand the roles elderly women in participation to maternal health, advices given to pregnant women and whether they influence obstetric complications among pregnant women in Limpopo province.

## Research methods and design

### Design

A qualitative, exploratory, descriptive and contextual research design was used (Creswell & Creswell 2018). This approach enables the researchers to explore and describe practices based on beliefs of elderly women influencing obstetric complications in Limpopo province. This research approach assisted the researchers to get rich information about the experiences, feelings and practices of elderly women influencing obstetric complications among pregnant women. The participants in Vhembe and Capricorn district of Limpopo province were able to articulate the information regarding their practices according to their beliefs and own indigenous knowledge.

### Setting

The study was conducted in the selected primary healthcare centres (PHC) of Vhembe and Capricorn district, Limpopo province, South Africa. The province consists of five districts, namely Capricorn, Mopani, Sekhukhune, Vhembe and Waterberg. The province is considered poor, with approximately 80% of people living in rural areas. Vhembe district has the largest population and highest number of pregnant women (Saving Mothers 2017) whereas Capricorn District has an escalating number of maternal mortality as reported by Saving Mothers (2017).

### Study participants and inclusion criteria

#### Population and sampling

Elderly women were chosen as they are the caregivers of pregnant women at home. Non-probability purposive sampling method was used to sample elderly women who met the criteria, accepted and were willing to share their roles. Purposive sampling was chosen for this study as it focussed on elderly women who are relevant participants to narrate their roles. Arrangements were done with the headman on the day of gathering and the researcher was given opportunity to address the elderly women about the study where the information sheet was read to them. Furthermore, appointments were made with the participants who were willing to participate and had consented. Inclusion criteria were elderly women aged 50–80 years whose daughter or daughter-in-law delivered in the past 5 years, mentally stable and living in the geographical area of the study site. Elderly women who were not mentally stable and had not consented to participate were excluded. A total number of six focus group discussions (FDGs) were conducted of which four FDGs had six members per group, one had four members and one had nine members to make a total number of 37 participants.

### Data collection

Unstructured questions for FGDs were used to collect data from December 2021 up to April 2022 at chief’s kraal. The question that guided the interview was, ‘you are elderly women, caregivers of the PW at home, *kindly share with me your roles and the advice that you give to pregnant women*’. Probing was done based on the participant’s response to encourage participants to bring out more information on the question explored. During data collection, permission was sought and granted by participants to use voice recorder to capture and record the information. This was done to make sure all the information given by the participants was captured. Codes were assigned to participants from FGD 1 to 6. Data saturation was reached at the fourth group of FGDs; however, the researcher went on with the interviews by adding another two FDGs to make sure no new information was coming in. The interview took 1–2 h for each group.

### Data management and analysis

Data were protected by employing a computer that locks and unlocks files with a secret pin. The researcher read the entire field notes taken and listened to audiotape recorder and wrote the transcript of group discussions per group. This involved transcribing and translating information from the audio recorder into English from the vernacular in written words assisted by a language expert. After transcribing, the researcher repeatedly read each transcript to familiarise herself with the data, simultaneously sorting similar and different ideas. Data analysis was done guided by Tech’s eight steps in the coding process described by Seopa (2021). The analysis was done by the researcher with the assistance of a team of authors collectively in a scheduled workshop. This involved reading of transcript several times to understand what elderly women said. Collected data were classified into categories and sub-categories using the actual words from the participants. Similar categories were compared and further allocated to overall categories. Finally, two themes and three sub-themes were developed and refined. An independent coder who was well-versed in the subject matter of the study was involved to ensure objectivity. Working separately, the independent coder and researchers produced a precise context for the data that were gathered. The interpretation of findings was based on researcher’s understanding about maternal health and integration of various literatures regarding the topic of study. Follow-up of the captured data was done to verify the information given by participants was indeed what they have said.

### Measures to ensure trustworthiness

As indicated by Polit and Beck (2020), credibility was ensured by remaining in the field for about 3–5 months engaging with the participants and as such, a trusting and mutual relationship was established between the interviewer and the participants by creating an environment where the participants would feel free to express themselves in their home language. The prolonged engagement was ensured by visiting the participants repeatedly to ensure that more time was invested, for about 1–2 h, as it was a FDG. Dependability of the study was ensured by recording all details regarding the FDGs and documentation for others to repeat the study with the same participants in a similar context. Conformability was ensured by listening to audio recorded as much as possible to verify interpretations, conclusions and recommendations. Transferability was ensured by giving clear detailed description, which involves the setting, participants and methods used to collect data for other researchers to come up with the same findings and conclusions.

### Ethical considerations

The study was approved, in 2020, by the Higher Degrees Ethics Committee and Research and Publications Committee of the University of Venda (reference no.: SHS/20/PDC/35/2809). Approval was received from the Department of Health Limpopo Province Research Ethics Committee and Vhembe and Capricorn district nurse managers of the clinics. Ethical considerations were adhered to and were in accordance to statutory ethical standards. Participants were assured, for the purpose of ethical issues, that participation was voluntary and that they can withdraw from participation at any time without any form of harm or penalty and no reward of participation to be awarded. Ethical principles were adhered to as the participants signed the consent form voluntarily, anonymity was observed as the researcher used code instead of real names to maintain confidentiality. This was done so that participants cannot be linked with the data. All participants participated until the end with no withdrawal. Records were kept safe in a passworded gadget and a copy was available to the researchers only.

## Results

Demographic data of participants are presented in Table 1, where six FDGs were conducted, four with six members per group and one group with four participants and another one with nine members.

**TABLE 1:** Age distribution of participants (*N* = 37).

Participants	Age (years)	*n*	Language	*n*
Elderly women	50–59	1	Tshivenda	28
	60–80	36	Sepedi	9

The sample consisted of 37 elderly women (focus group) who offered care to PW at home (refer to Table 1).

### Theme 1: Practices based on beliefs of elderly women

Theme 1 emerged from data indicating various practices based on beliefs that elderly women gave to PW under their care at home. Two sub-themes identified from the theme were practices during the pregnancy period and during the postnatal period (Table 2).

**TABLE 2:** Themes and sub-themes.

Theme		Sub-theme
1.	Practices based on beliefs of elderly women	1.1 Practices during the pregnancy period 1.2 Practices during the postnatal period
2.	Need of elderly women to participate in maternal health practices	2.1 Need for training

Each sub-theme was discussed separately under theme of practices based on the beliefs of elderly women.

#### Sub-theme 1.1: Practices during the pregnancy period

Data analysis revealed that elderly women played a role in installing some of the practices for PW during pregnancy period based on their beliefs. These practices delay pregnant women in seeking early medical intervention. Some practices include consulting traditional healers or religious leaders whenever PW under their care and support receive negative information about their pregnancies. Participants admitted visiting traditional healers after receiving unbearable information from the PW under their care after a visit to the doctor. In support of this, participants articulated:

‘… maybe she is possessed so I consult with traditional people such as the traditional healers or the prophets. They can assist the pregnant woman and get deliverance afterwards because sometimes the problem might be caused by spirits …’ (FGD4, Participant 4, Female)

‘… I [*mother-in-law*] will take her [*pregnant woman*] to a traditional healer, I just assume that let me take her to a traditional healer so that he can assist her to open up a way for the baby …’ (FGD6, Participant 2, Female)

What Participant 4 shared earlier shows the extent to which abnormal experiences are easily associated with evil spirits. To show how firmly they believed that evil spirits were always at work to destroy the unborn baby, Participant 4 proceeded further to articulate that:

‘The pregnant women can be assisted and receive the deliverance because the spirits that are caused by people can make the pregnant woman fail to give birth normally whereas the pregnant woman can deliver normally under normal circumstances …’ (FGD4, Participant 4, Female)

The study findings revealed that culturally, the issue of pregnancy is centred around the family wherein no one outside the family should be told, especially in the first trimester. It is believed that these practices are followed to conceal and protect the unborn baby from bewitched results in miscarriage. The following quotes support this:

‘… when one is pregnant you have to hide it because other people are jealous to an extent that they will cause you to have something that is not good to you …’ (FGD6, Participant 3, Female)

‘Pregnant woman must attend antenatal clinic from 4 months of gestation in order for her to be sure of pregnancy and to hide the pregnancy to avoid to bewitched if she discloses pregnancy at an early stage …’ (FGD1, Participant 1, Female)

The study revealed that elderly women play a role in sharing information about practices based on their beliefs to PW regarding the issue of registering for ANC. One key issue that emerged from the findings is that elderly women were comfortable with PW registering for ANC only when the pregnancy was at 16 weeks (4 months) gestation and above. This was motivated by the view that pregnancy from 16 weeks of gestation would have passed the stage where a PW can experience miscarriages that can put shame on the family if known by other members outside the family. Culturally, elders use their indigenous knowledge of believing in witchcraft; they think that there are jealous people who can get rid of that pregnancy. These practices contributed to late registration of ANC, which may result in negative pregnancy outcomes. Participants expressed their comfortability with encouraging PW to register with ANC post-miscarriage period by articulating that:

‘Pregnant woman must attend antenatal clinic from 4 months of gestation in order for her to be sure of pregnancy and to avoid to bewitched if she discloses pregnancy at early stage …’ (FGD1, Participant 1, Female)

‘She has to go and book for ANC when she is three months pregnant …’ (FGD6, Participant 1, Female)

The study’s findings revealed that elderly women encouraged PW to adhere to traditional practices for preparing for natural birth. Elderly women would therefore take a PW who is near term to a traditional healer to perform rituals to open the birth canal for a woman to give birth naturally and prevent difficult labour. This is supported by the following quotes:

‘What we can do is to do tsemo [*steaming*] to the pregnant woman where we prepare the fire and let her stand so as to open the birth canal …’ (FGD5, Participant 6, Female)

‘When she is at term, from nine months we take her to a traditional healer and she must start with tsemo [*steaming*] until she gives birth …’ (FGD5, Participant 6, Female)

This study revealed that PW were told to refrain from sexual acts with their husbands as soon as their pregnancy reached 8 months of gestation. One participant articulated the following:

‘… yes the other thing is that a pregnant woman when she approaches eight months of pregnancy I tell her that she must stop to have sexual intercourse because the head of the baby is down and the semen will stick on the head of the baby and it is not a good thing, especially to the old women who will assist the mother on the care of the baby after delivery …’ (FGD6, Participant 3, Female)

#### Sub-theme 1.2: Practices during the postnatal period

The findings in this study revealed practices during the postnatal period that elderly women perform for BFMs under their care. The elders believe in using traditional herbs to treat some minor ailments that can arise during the postnatal period. Supporting this, the following quotes were articulated:

‘When the mother has given birth, she will usually complain of tshikangala [*after pains*] and it is more painful compared to labour pains, so I boil [*munawa*] certain leaves and let the mother drink to treat tshikangala and indeed the pain will be disappear …’ (FGD2, Participant 4, Female)

‘… we cook certain leaves [*dikgalo*] and pat the abdomen with it in order to take out the remaining blood because at the hospital they inject the woman soon after delivery [*with oxytocin*] so as to stop bleeding, and that blood will kill her because she is supposed to bleed more up to three months …’ (FGD3, Participant 9, Female)

This study revealed that for a newborn to come out from the isolation room for the first time, some rituals must be performed to protect the baby from certain sicknesses. Elderly women at home are seen as the person who facilitates the process of rituals by a traditional healer. Participants articulated the following:

‘It is about the newborn baby, rituals should be performed to the baby. To strengthen the bones and the stomach of the newborn, to prevent frequent passing of loose stools …’ (FGD2, Participant 1, Female)

‘… the baby must remain inside the yard until such time when the baby is growing to an extent that other can be able to hold her is when the ritual is performed again for the baby to go outside of the gate …’ (FGD4, Participant 4, Female)

This study revealed that during the postnatal period, mothers were encouraged to feed the baby with soft porridge immediately after birth, believing that the baby would be born hungry. Participants articulated the following:

‘Yes we used to deliver a very tiny baby but soon after delivery the baby should be fed …’ (FGD2, Participant 2, Female)

‘Newborn should be given feeds (s oft porridge) immediately after delivery because the baby will be hungry as the mother was getting a small amount of food during pregnancy …’ (FGD1, Participant 2, Female)

### Theme 2: Need of elderly women to participate in maternal health practices

Theme 2 that emerged from the data analysis is the need of elderly women to participate in maternal health practices. The theme has one sub-theme that is, need for training (Table 2).

#### Sub-theme 2.1: Need for training

The study revealed that elderly women as caregivers of PW need training as they have their own way of caring for PW using indigenous information acquired from their ancestors and life experience. Some indigenous information is believed to cause delays for PW in seeking early medical attention. Participants understand that they provide care to the PW in an unprofessional manner and that they need to be empowered to save the lives of both the mother and child. This is supported by the following quotes:

‘… nurses must come and teach us …’ (FGD3, Participant 1, Female)

‘… they [*nurses*] can come to teach us not to control pregnant woman on how to eat and what not to eat …’ (FGD3, Participant 4, Female)

‘For us as caregivers at home, nurses are the ones who are supposed to teach us …’ (FGD6, Participant 3, Female)

## Discussion

The study found that elderly women play significant roles in the life of PW, newborn and BFMs. The study findings revealed two essential roles that are played by elderly women in maternal health: (1) Practices are based on beliefs of elderly women during pregnancy and postnatal period, and (2) Need for elderly women to be trained to participate in maternal health practices. While playing these roles, elderly women intentionally or unintentionally influence the attitudes, habits and beliefs of PW towards seeking medical assistance from health care facilities in their vicinities. According to the attestations from elderly women, it is their duty to advise PW on maternal health issues to safeguard the women and the foetus or the child. Furthermore, these practices are based on religious, traditional and cultural beliefs. Unfortunately, out of ignorance or pressured by economic conditions, PW just follow the practices blindly as some are not aware of the dangers that might befall them until it is too late to seek medical assistance from local health facilities. Although some of the practices could work in some instances, they seem to lack the merit required to safeguard the potential victims when there are complications.

### Practice based on beliefs of elderly women during pregnancy and postnatal period

The findings of this study indicated that elderly women played a role in influencing some of the practices for PW during pregnancy period based on their beliefs. These practices include consulting traditional healers or religious leaders whenever PW under their care and support receive negative information about their pregnancies. Elderly women would believe that negative information could have been caused by witchcraft which makes them take PW to the traditional healers to clear the negative information. These practices could cause delay in PW seeking medical intervention resulting in obstetric complications. This is supported by Seopa (2021), who states that traditional medicines were used to protect pregnant women against witchcraft and miscarriages. Similarly, Ozioma and Chinwe (2019) state that spiritual cleansing is a process where a sick or pregnant person is subjected to a bath by water or animal blood as a religious or cultural activity.

The study findings revealed that elderly women told the PW not to tell anyone outside the family about the pregnancy before it passes the stage of miscarriage (3 months). Culturally, the issue of pregnancy is centred around the family wherein no one outside the family should be told, especially during the first trimester. Moreover, it is believed that these practices are done to conceal and protect the unborn baby from being bewitched resulting in miscarriage. This was supported by Lang-Baldé and Amerson (2018) who emphasised that pregnancy should be kept secret and must not be revealed until such time it is no longer possible to hide it. However, this increases maternal and perinatal morbidity and mortality rates as it reduces the minimum number of eight ANC contacts as recommended by WHO (2016) for the purpose of minimising obstetric complications. Furthermore, findings revealed practices based on tradition where elderly women play a role in PW registering for ANC. One key issue that emerged from the findings is that elderly women were comfortable with PW registering for ANC only when the pregnancy was at 16 weeks (4 months) gestation and above as it is believed that the women by then would have passed the period of miscarriage. In agreement, Ragolane (2017) emphasises that it is not customary for Africans to publicise their pregnancy too early because of fear of being bewitched by jealous people.

The study findings revealed practices based on tradition where elderly women encouraged PW to adhere to traditional practices when they are near term to prepare them to give birth naturally. Elderly women would take a PW who is near term to a traditional healer to perform rituals which they believed opens the birth canal for a woman to give birth naturally and prevent difficult labour. These practices based on tradition could delay a woman to seek medical intervention leading to obstetric complications especially in case where the woman is having cephalo-pelvic disproportion (CPD). This is supported by Aljofan and Alkhamaiseh (2020) and Thipanyane et al. (2022), who state that the use of traditional health medicine makes the PW have a hassle-free pregnancy to shorten labour and improve breast feeding.

In addition, PW were told to refrain from sexual acts with their husbands as soon as their pregnancy reached 8 months of gestation. This finding concurred with Buser (2019), who stated that in Lundazi and Mansa districts, Zambia, a woman is discouraged from sleeping with their husband at 8 months of gestation to avoid the baby being born with sperm on the head; it is believed that vernix caseosa is sperm. The finding contrasts with WHO (2016) that sexual intercourse during pregnancy is not known to be associated with any adverse outcomes.

Furthermore, elders believe that using traditional herbs can treat some minor ailments that can arise during the postnatal period (Aljofan & Alkhamaiseh 2020; Thipanyane et al. 2022). This study revealed that for a newborn to come out from the isolation room for the first time, some rituals must be performed to protect the baby from certain sicknesses. Hence, elderly women at home are seen as the persons who facilitate the process of rituals by a traditional healer. This finding is consistence with the finding reported by Buser (2019), as cited by Masilo (2022), from a study conducted in Limpopo, South Africa. The study reported there is a traditional medicine used to treat childhood illness called *Dupa* which might cause harm to an infant if not properly used as its direction for use is not clear. This confirms that certain rituals are done to the babies involving traditional medicine as encouraged by elderly women who seemed to have a final word in the family. The study further revealed that the practices during the postnatal period indicated that mothers were encouraged to feed the baby with soft porridge immediately after birth, believing that the baby would be born hungry. This finding is in agreement with Buser (2019), who stated that women in Mansa district, Zambia, introduce traditional porridge as early as 1 month of life. The porridge was mixed with herbs to protect the baby from childhood sicknesses. In contrary to Li et al. (2021), Issaka et al. (2014) and WHO (2015) stated that a newborn baby should be breastfed exclusively for 6 months or formula-fed exclusively if there is a contra-indication to breastmilk or in case the baby is an orphan.

### Need for elderly women to be trained to participate in maternal health practices

The study findings further revealed that elderly women as the ones who are always monitoring PW at home, need training as they have their own way of caring for PW using indigenous information acquired from their ancestors and life experience. Some indigenous information is believed to cause delays for PW in seeking early medical attention leading to obstetric complications. Participants of this study understand that they provide care to the PW in an unprofessional manner. Therefore, they need to be empowered to save the lives of both the mother and child. This finding is supported by Jama et al. (2024) and Mogawane (2014) who state that community involvement and active participation should be strengthened on the issues relating to ANC, thereby conducting training workshops and traditional awareness campaigns for THPs, church leaders, elders and the community at large. In agreement with Awolayo (2019), who established that community mobilisation and awareness and training of TBAs who may also serve as maternal delegates within the community is recommended in order to discourage or reduce unsafe practices and promote maternal health care services.

Elderly women are expected to care for the PW on various aspects of maternal health. Most importantly, seeking professional medical assistance as early as possible is seen to be a matter of concern to minimise obstetric complications. However, this does not happen because when something goes wrong, elderly women whose views are still stuck in old traditions, religious and cultural beliefs either blame the PW and BFMs for not listening to them or see complications as the works of witchcraft. These findings show that without proper training on professional maternal health, elderly women, who are the immediate persons, will continue providing inaccurate and outdated practices that endangers PW. This, in turn, will accelerate maternal mortality rates, as the study revealed that practices based on beliefs of elderly women cause delay for PW and BFMs to seek medical assistance, practices such as taking the women to the traditional healer to perform some ritual and to the religious leader for prayer while in labour so that they could deliver normally especially those who were told that they would deliver through caesarean section. Such practices put the pregnant women in danger as they increase complications, where some have even died on arrival at the health care facilities. These practices will, however, continue; hence, this study revealed the importance of training the elderly women in order for them to refrain from these cultural practices.

### Strength and limitations

The results of the study are important for improving the likelihood that maternal death rates will be reduced in the Vhembe and Capricorn districts by training elderly women to participate in maternity health practices.

The hospitals in the sampled districts were not included in this study; it was limited to the clinics in the Vhembe and Capricorn districts. Only elderly women between the ages of 50 and 80 years were interviewed for the contextual study. The Vhavenda and Bapedi groups constituted the majority of the thoughts and opinions used in the study. The coronavirus disease 2019 (COVID-19) pandemic had an impact on the study’s timing as well as the quantity of participants and time spent in contact with them.

### Recommendations

To save the life of pregnant women from socio-cultural practices of elderly women, the study recommended the following:

- Understanding the roles and advice given to PW by elderly women will assist in redirecting the necessary information so that it can be properly and effectively used by explaining the myths and misinterpretations thereof.
- Developing a training programme for elderly women in participation to maternal health practices to minimise obstetric complications caused by socio-cultural practices. Therefore, elderly women should be trained with the aim to minimise obstetric complications and improvement in the reduction of maternal mortality.

## Conclusion

Based on the discussion, the study concludes that socio-cultural practices contribute to obstetric complications. This is because elderly women were seen to be playing a significant role as they acted as advisers to PW who provided care and support during pregnancy and breastfeeding period. However, all roles were based on traditional, cultural and/or religious beliefs, which influenced the PW to delay in seeking medical health from healthcare facilities. The roles played by the elderly women were essential in maternal health in general but needed to be improved to synchronise with those provided at the medical care facilities. In agreement, training programme for elderly women in participation to maternal health practices was developed to minimise obstetric complications and reduction of maternal morbidity and mortality.
